# Preclinical and clinical evaluation of German-sourced ONC201 for the treatment of H3K27M-mutant diffuse intrinsic pontine glioma

**DOI:** 10.1093/noajnl/vdab169

**Published:** 2021-11-19

**Authors:** Ryan J Duchatel, Abdul Mannan, Ameha S Woldu, Tom Hawtrey, Phoebe A Hindley, Alicia M Douglas, Evangeline R Jackson, Izac J Findlay, Zacary P Germon, Dilana Staudt, Padraic S Kearney, Nathan D Smith, Kate E Hindley, Jason E Cain, Nicolas André, Andres Morales La Madrid, Brett Nixon, Geoffry N De Iuliis, Javad Nazarian, Kathleen Irish, Frank Alvaro, David D Eisenstat, Alexander Beck, Nicholas A Vitanza, Sabine Mueller, Jonathan C Morris, Matthew D Dun

**Affiliations:** 1 Cancer Signalling Research Group, School of Biomedical Sciences and Pharmacy, College of Health, Medicine and Wellbeing, University of Newcastle, Callaghan, New South Wales, Australia; 2 Hunter Medical Research Institute, New Lambton Heights, New South Wales, Australia; 3 School of Chemistry, University of New South Wales, Sydney, New South Wales, Australia; 4 Jewells Medical Centre, Jewells, New South Wales, Australia; 5 Analytical and Biomolecular Research Facility, Advanced Mass Spectrometry Unit, University of Newcastle, Callaghan, New South Wales, Australia; 6 Sash Small Animal Specialist Hospital, Tuggerah, New South Wales, Australia; 7 Hudson Institute of Medical Research, Clayton, Victoria, Australia; 8 Department of Molecular and Translational Science, Monash University, Clayton, Victoria, Australia; 9 Department of Pediatric Oncology, La Timone Children’s Hospital, AP-HM, Marseille, France; 10 SMARTc Unit, Centre de Recherche en Cancérologie de Marseille, Inserm U1068, Aix Marseille Univ, Marseille, France; 11 Laboratory of Developmental Cancer, Institut de Recerca Sant Joan de Déu, Barcelona, Spain; 12 Department of Oncology, Hospital Sant Joan de Déu, Barcelona, Spain; 13 Neuro-Oncology Unit, Hospital Sant Joan de Déu, Barcelona, Spain; 14 Reproductive Science Group, College of Engineering, Science and Environment, University of Newcastle, Callaghan, New South Wales, Australia; 15 Children’s National Medical Center, Washington, District of Columbia., USA; 16 University Children’s Hospital Zurich, Zurich, Switzerland; 17 John Hunter Children’s Hospital, New Lambton Heights, New South Wales, Australia; 18 Children’s Cancer Centre, The Royal Children’s Hospital Melbourne, Parkville, Victoria, Australia; 19 Neuro-Oncology Laboratory, Murdoch Children’s Research Institute, Parkville, Victoria, Australia; 20 Department of Paediatrics, University of Melbourne, Parkville, Victoria, Australia; 21 Center for Neuropathology, Ludwig Maximilian University of Munich, Munich, Germany; 22 Ben Towne Center for Childhood Cancer Research, Seattle Children’s Research Institute, Seattle, Washington, USA; 23 Division of Pediatric Hematology/Oncology, Department of Pediatrics, Seattle Children’s Hospital, Seattle, Washington, USA; 24 Department of Neurology, Neurosurgery and Pediatrics, University of California, San Francisco, California, USA

**Keywords:** diffuse intrinsic, pontine glioma, diffuse midline glioma, ONC201

## Abstract

**Background:**

Diffuse intrinsic pontine glioma (DIPG) is a fatal childhood brainstem tumor for which radiation is the only treatment. Case studies report a clinical response to ONC201 for patients with H3K27M-mutant gliomas. Oncoceutics (ONC201) is only available in the United States and Japan; however, in Germany, DIPG patients can be prescribed and dispensed a locally produced compound—ONC201 German-sourced ONC201 (GsONC201). Pediatric oncologists face the dilemma of supporting the administration of GsONC201 as conjecture surrounds its authenticity. Therefore, we compared GsONC201 to original ONC201 manufactured by Oncoceutics Inc.

**Methods:**

Authenticity of GsONC201 was determined by high-resolution mass spectrometry and nuclear magnetic resonance spectroscopy. Biological activity was shown via assessment of on-target effects, *in vitro* growth, proliferation, and apoptosis analysis. Patient-derived xenograft mouse models were used to assess plasma and brain tissue pharmacokinetics, pharmacodynamics, and overall survival (OS). The clinical experience of 28 H3K27M+ mutant DIPG patients who received GsONC201 (2017–2020) was analyzed.

**Results:**

GsONC201 harbored the authentic structure, however, was formulated as a free base rather than the dihydrochloride salt used in clinical trials. GsONC201 *in vitro* and *in vivo* efficacy and drug bioavailability studies showed no difference compared to Oncoceutics ONC201. Patients treated with GsONC201 (n = 28) showed a median OS of 18 months (*P* = .0007). GsONC201 patients who underwent reirradiation showed a median OS of 22 months compared to 12 months for GsONC201 patients who did not (*P* = .012).

**Conclusions:**

This study confirms the biological activity of GsONC201 and documents the OS of patients who received the drug; however, GsONC201 was never used as a monotherapy.

**Key Points**

- The German-sourced ONC201 (GsONC201) is the active angular isomer.
- GsONC201 is formulated as a free base while Oncoceutics (ONC201) is a dihydrochloride salt.
- No difference in the anti-DIPG properties were seen when comparing ONC201 and GsONC201.
- OS of patients using German-sourced ONC201 is 18 months (*P* = .0007).
- OS of GsONC201 patients who had reirradiation was 22 months (*P* = .012).

**Importance of the Study**

To date, all clinical trials for children diagnosed with DIPG have failed to increase median overall survival, which remains 9–11 months. Recently, clinical studies testing ONC201 in glioblastoma and in DIPG case studies suggest a therapeutic benefit for patients who harbor H3K27M mutations, encouraging clinical trials in DIPG where the mutation is seen in more than 80% of cases. However, access to ONC201 is limited to patients in the United States and Japan. Commercial access to a synthesized version of ONC201 (GsONC201) is available in Germany; however, no information is available about its formulation, bioavailability, or efficacy, compared to ONC201 (Oncoceutics). We examined GsONC201 and show that it is the active angular isomer, formulated as a free base as opposed to ONC201 which is a dihydrochloride salt, and increased survival of a DIPG PDX model. We assessed the clinical experience of 28 patients receiving GsONC201 and report a median overall survival of 18 months.

Diffuse intrinsic pontine glioma (DIPG) is the most aggressive and lethal form of childhood cancer, with a median overall survival (OS) 9–11 months^1,2^ and less than 10% of patients surviving 2 years postdiagnosis.^3^ More than 80% of cases harbor a lysine 27 to methionine mutation in histone H3 encoding genes [H3K27M, *HIST1H3B* (H3.1) or *H3F3A* (H3.3)].^1^ Over 50 years of clinical trials have failed to improve DIPG survival,^4–6^ and treatment remains palliative radiotherapy.^7^

Described as a dopamine receptor D2 (DRD2) selective antagonist,^8^ the blood–brain barrier (BBB) penetrant imipridone, ONC201, is also a potent agonist of the mitochondrial Caseinolytic protease P (ClpP).^9,10^ When activated by ONC201, ClpP drives degradation of mitochondrial respiratory chain enzymes, such as the succinate dehydrogenase subunits A and B (SDHA and SDHB), triggering p53-independent apoptosis and cancer-selective cell death.^11^ ONC201 case studies and expanded access programs show objective clinical responses for pediatric patients diagnosed with DIPG or diffuse midline glioma (DMG) and harboring H3K27M mutations; in some cases, resulting in complete regression of the primary tumor. Moreover, patients who initiated adjuvant ONC201 following radiation remained progression-free for at least 53 and 81 weeks.^12,13^ These results encouraged new clinical trials to test ONC201 in pediatric patients diagnosed with H3K27M-mutant DMG, including DIPG (NCT03416530). However, access to these trials is limited almost exclusively to patients in the United States or Japan.

Signals of efficacy for patients diagnosed with DIPG receiving ONC201 have resonated throughout the international DIPG community, partly due to an alternative access pathway available under German compassionate care regulations. However, published details regarding the chemical characteristics of the German synthesized version of ONC201 (henceforth referred to as “GsONC201”) are lacking, thus highlighting the critical necessity to characterize the authenticity of GsONC201, particularly given earlier reports of the availably of an inactive [4,3-d] linear isomer of ONC201.^14^ Consequently, in this study, we have characterized the formulation, isometry, *in vitro* biochemical and anti-DIPG properties, in vivo pharmacokinetics (PK), pharmacodynamics (PD), and DIPG patient-derived xenograft (PDX) mouse model survival, as well as document the clinical experience of DIPG patients receiving GsONC201, using capsules donated by 8 international families of children diagnosed with DIPG from 2017 to 2020.

## Materials and Methods

Detailed materials and methods are provided in Supplementary Material.

### Drugs

ONC201 was provided directly by Oncoceutics Inc under a materials transfer agreement. Twenty unopened capsules of GsONC201 were donated by 8 families of children diagnosed with DIPG, 2017–2020, purchased in Germany following a confirmed diagnosis of DIPG. All patients had not previously enrolled in a clinical trial.

### High-Resolution Tandem Mass Spectrometry

High-resolution tandem mass spectrometry was performed on GsONC201 and ONC201 (Oncoceutics) using direct infusion into an accurate mass Thermo Scientific Q Exactive Plus mass spectrometer (Thermo Fisher Scientific). Samples were resuspended in 1 mL of dimethyl sulfoxide (DMSO), diluted 1 in 100 in 50:50 methanol:acetonitrile/0.1% formic acid, and analyzed using a resolution of 140 000 at 200 m/z for both MS1 and MS2. Data analysis was performed using Mass Frontier 7.0 (Thermo Fisher Scientific).

### Nuclear Magnetic Resonance Spectroscopy

Nuclear magnetic resonance (NMR) spectroscopy was performed using either a Bruker Avance 300 (300.13 MHz, ^1^H; 75.5 MHz, ^13^C) or an Avance III 400 (400.13 MHz, ^1^H; 100.6 MHz, ^13^C) with or without a Prodigy cryoprobe CPPBBO. NMR spectra were processed using TopSpin 3.5 software (Bruker). Chemical shifts are expressed in parts per million (ppm) on the δ scale. Chemical shifts in CDCl_3_ were referenced relative to CHCl_3_ (7.26 ppm) for ^1^H NMR and CDCl_3_ (77.16 ppm) for ^13^C NMR and chemical shifts in (CH_3_)_2_SO were referenced relative to (CH_3_)_2_SO (2.50 ppm) for ^1^H NMR and (CD_3_)_2_SO (39.52 ppm) for ^13^C NMR.

### In Vitro Assessment of Anti-DIPG Effects

DIPG cell lines were provided as a generous gift from Prof Michelle Monje and maintained as described.^15^ Cell growth and proliferation were determined as described,^16^ using 2.5 × 10^4^ cell/well, plated the day before the addition of ONC201 or GsONC201 to allow for neurosphere formation. Drug sensitivity was assessed over 96 h. Annexin V/PI assays were performed as described,^17^ using 5 μM ONC201 or GsON201 over 72 h. Western blotting was performed as described.^18^

### Drug Docking

Assessment of ONC201 and GsONC201 into their putative targets was performed as described,^17^ using GOLD Protein-Ligand Docking Software (version 2020.2.0).^19,20^

### Animal Studies

All *in vivo* studies were approved by the University of Newcastle Animal Care and Ethics Committee (#A-2019-900 and #A-2020-004).

### PK, PD, and In Vivo Efficacy

Pharmacokinetic studies were performed using ONC201 and GsONC201 diluted in 1% methylcellulose/0.2% Tween 80, 15 mg/kg, given by gavage to 8-week-old BALB/c Nude mice. Mice were treated with omeprazole (1.5 mg/kg/daily) for 7 days prior to administration of either ONC201 or GsONC201. After 1 h, mice were sacrificed by CO_2_ euthanasia. Immediately following euthanasia, blood was extracted via cardiac puncture, and stomachs and brains were collected. Blood plasma was separated via standard centrifugation techniques and tissues homogenized prior to freezing and multiple reaction monitoring mass spectrometry. Pharmacodynamic analysis of brainstem H3K27M+ SU-DIPG-VI and control prefrontal cortex tissues following oral treatment with vehicle, ONC201 and GsON201 125mg/kg and sacrificed 48 h after treatment. Hematoxylin and eosin staining (H&E) and Ki67 staining of brainstem SU-DIPG-XIIIP* tumor tissue following 2 weeks of treatment with vehicle, ONC201 or GsON201 125 mg/kg. Kaplan–Meier survival analysis was performed using SU-DIPG-XIIIP* treated with vehicle, ONC201 or GsON201 125 mg/kg every 5 days.

### Clinical Experience

Written informed consent was obtained from the guardian of each patient who received GsONC201 between 2017 and 2020 (HNE HREC #AU202105-02). The de-identified patients were managed by the clinical authors listed herein. Patients were not on a clinical trial. Survival data were collected until April 20, 2021 (Supplementary Table 1). Data from patients with confirmed H3K27M status (genomics/IHC) were used in determining survival probabilities.

## Results

### Analysis of German-Sourced ONC201 Reveals Active Isometry but Alterations in Formulation

**Figure 1.**
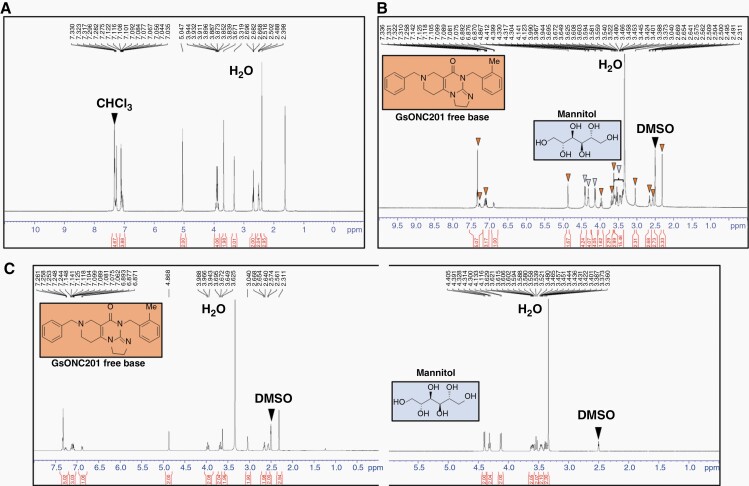
Nuclear magnetic resonance (NMR) spectroscopy of German-sourced ONC201 (GsONC201) revealed the active component is the “angular isomer,” formulated as a free base, and the formulation contains mannitol. (A) ^1^H NMR spectrum of soluble component of GsONC201 capsules in CDCl_3_ showed that the active component is present as the free base (GsONC201), which matches the literature reports values for the bioactive “angular structure”.^21^ (B) ^1^H NMR spectrum of GsONC201 capsules in DMSO-d_6_ confirms that it is formulated as the free base (structure included inside the orange box, with the peaks marked with orange arrowheads matching the data reported by Oncoceutics). The GsONC201 formulation also contains mannitol (C_6_H_8_(OH)_6_)—structure included inside blue box—the peaks marked with blue arrowheads match the reported data in approximately a 1:2 molar ratio based on the integration of peaks of mannitol:GsONC201. (C) ^1^H NMR spectra in DMSO-d_6_ of active component of GsONC201 and mannitol after separation using differential solubility in chloroform.

High-resolution tandem mass spectrometry revealed that GsONC201 has an identical chemical composition (C_24_H_26_N_4_O_1_) and molecular weight as Oncoceutics ONC201 (Supplementary Figure 1A and B) and is comparable to the results of Wagner et al.^14^ (Supplementary Figure 1A and B). Previous NMR spectroscopy structural analyses showed ONC201 harbors an angular [3,4-e] structure, rather than a linear [4,3-d] isomer mistakenly reported as the original ONC201 structure in 1973. Jacob et al.^21^ re-synthesized both the angular and linear isomers and confirmed that only the angular isomer exhibits the biological anticancer activity associated with ONC201. However, which isomer was present in GsONC201 remained unknown. NMR spectroscopy showed GsONC201 is the active, angular [3,4-e] structural isomer (Figure 1). However, GsONC201 is formulated as a free base rather than the dihydrochloride salt manufactured by Oncoceutics (Figure 1). The ^1^H NMR spectrum showed the GsONC201 is formulated to contain mannitol (Figure 1B and C). Mannitol (C_6_H_8_(OH)_6_) is an isomer of sorbitol and found to be present in the formulation in a 1:2 molar ratio (mannitol:GsONC201). In contrast to GsONC201, the formulation of ONC201 is a dihydrochloride salt (Supplementary Figure 2A) and insoluble in CDCl_3_.^14^ Given the differing solubilities of GsONC201 and mannitol, we separated each component and performed NMR in DMSO-d_6_ which resolved each independently (Figure 1C). Given that no spectroscopic data have been published for the salt, ONC201 was treated with base to convert it to the free amine, enabling us to confirm that the ^1^H NMR spectrum in CDCl_3_ of this free base matches the literature^21^ (Supplementary Figure 3B).

### Free Base GsONC201 Showed Analogous In Vitro Efficacy and Biochemical Activity to Oncoceutics ONC201

**Figure 2.**
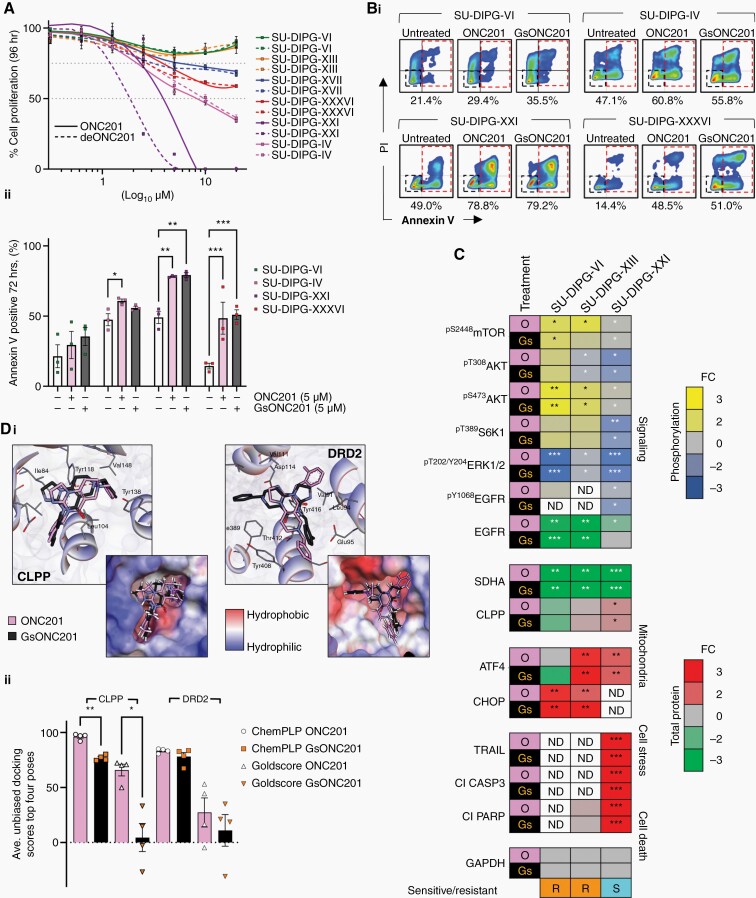
In vitro DIPG apoptosis is dependent on SDHA degradation following either ONC201 or German-sourced ONC201 (GsONC201) treatment. (A) Protonated ONC201 and free base German-sourced ONC201 (GsONC201) showed similar antiproliferative effects in DIPG cell lines: SU-DIPG-IV, SU-DIPG-XXI, SU-DIPG-XXXVI, SU-DIPG-VI, SU-DIPG-XIII, and SU-DIPG-XVII. (Bi) Cytotoxicity was assessed via annexin V/PI staining and flow cytometry following 72-h treatment with 5 μM ONC201 and GsONC201 (Bii) and compared with vehicle controls (Student’s *t*-test). (C) Both ONC201 (O; pink) and GsONC201 (Gs; black), activated CLPP to sequester SDHA, and modulate the activity of the mTOR/AKT signaling pathways. DIPG cell lines sensitive (S; aqua) to ONC201 or GsONC201 showed increased TRAIL expression and induced cell death, cleaved PARP and CASP3, whereas resistant DIPG cells lines (R; orange) showed no change in the activity in programmed cell death proteins (FC = fold change, yellow = increased phosphorylation, blue = decreased phosphorylation, red = increased protein expression or cleavage (Cl), green = decreased protein expression, ND = not determined; Student’s *t*-test used to determine significance between treated vs untreated samples, *P < .05, **P < .01, ***P < .001). (Di) Docking of both the implicit protonated ONC201 (pink structure) and free base ONC201 (black structure) into the defined binding pocket of CLPP (co-crystal structure of CLPP and ONC201 [PB: 6DL7; ONC201]), revealed that protonated ONC201 species (at N1, pKa = 8.1) is likely to have enhanced binding, indicated by the higher “fitness” scores calculated, in comparison to free base ONC201. This enhanced binding is facilitated by an H-bond interaction with the protonated N1 of ONC201 and the tyrosine 118 residue in the binding pocket. Docking of protonated ONC201 (pink) and GsONC201 (black) into the defined binding pocket of DRD2 did not show significant variation. (Dii) Quantification of unbiased docking scores of protonated and free base forms of ONC201 into CLPP and DRD2 (Student’s *t*-test used to determine the significance of ONC201 vs GsONC201 docking scores based on n = 4 top docking poses, *P < .05, **P < .01).

Having identified slight structural differences in free base GsONC201 compared to Oncoceutics ONC201, and in view of the paucity of preclinical data for ONC201 for the treatment of DIPG, we assessed the antitumor efficacy of both chemicals using patient-derived DIPG cell lines. Growth and proliferation assays performed on patient-derived DIPG cells lines (n = 6) harboring mutations that cover the spectrum of known DIPG mutations,^22^ which showed similar levels of sensitivity to each chemical (Figure 2A). DIPG cell lines SU-DIPG-XXI (H3.1K27M, *ATM*, and *MCL1* mutant), SU-DIPG-IV (H3.1K27M and *PIK3CA* mutant), and SU-DIPG-XXXVI (H3.1K27M, *PIK3R1*, and *ACVR1* mutant) were extremely sensitive to both chemicals *in vitro*, whereas SU-DIPG-VI (H3.3K27M, *MCL1*, and *TP53* mutant) and SU-DIPG-XIII (H3.3K27M and *TP53* mutant) showed reduced sensitivity to both ONC201 and GsONC201 and failed to reach IC_25_. SU-DIPG-VI and SU-DIPG-XIII are known to be highly aggressive postradiation autopsy patient-derived DIPG cell line models, reflected by the poor survival of both patients from which they were established.^22^ Given the dramatic difference in the *in vitro* antiproliferative effects, we assessed apoptosis via flow cytometry and annexin V staining, confirming ONC201 or GsONC201 to be both antiproliferative and cytotoxic in SU-DIPG-XXI, SU-DIPG-IV, and SU-DIPG-XXXVI (Figure 2Bi and ii). Analogous to proliferation analysis, SU-DIPG-VI did not show a significant increase in annexin V staining following ONC201 or GsONC201 treatment, however, did show a nonsignificant trend (Figure 2Bi and ii).

Given the range of sensitivities between DIPG cell lines to either ONC201 or GsONC201, we investigated whether free base GsONC201 would affect its biochemical mechanism of action. Treatment of the highly sensitive DIPG cell line SU-DIPG-XXI with either ONC201 or GsONC201, ablated phosphorylation of ^pT202/Y204^ERK, ^pT308/S473^Akt, and ^pS2448^mTOR (Figure 2C, Supplementary Figure 4A). Furthermore, both ONC201 and GsONC201 induced endogenous ATF4-regulated TRAIL expression in SU-DIPG-XXI and robust cell death through the activation of the cell death receptor apoptotic signaling cascade—CASP3 and PARP^23,24^ (Figure 2C, Supplementary Figure 4B). Intriguingly, both ONC201 and GsONC201 decreased phosphorylation of ^pY1068^EGFR in SU-DIPG-XXI (Figure 2C, Supplementary Figure 4A), whereas in the more resistant cell lines SU-DIPG-VI or SU-DIPG-XIII, both chemicals decreased total EGFR protein expression.

Importantly, in all tumor cell lines investigated, both ONC201 and GsONC201 appeared to act as an agonist of the mitochondrial protease ClpP,^9,10^ independent of their antiproliferative and anticytotoxic effects (Figure 2A–C, Supplementary Figure 4A). Proteolytic degradation of SDHA and mitochondrial dysfunction^9,10^ appear to be the major drivers of cell death in sensitive DIPG cell lines, whereas less sensitive cells showed increased phosphorylation of ^pS473^Akt and ^pS2448^mTOR signaling following treatment (Figure 2A–C, Supplementary Figure 4A). Hence, no change in phosphorylation of ^pT389^S6K1 was seen with either treatment in less sensitive cell lines (Figure 2C, Supplementary Figure 4A). Unsurprisingly, given the reduced antigrowth and apoptosis in SU-DIPG-VI or SU-DIPG-XIII, there was no increase in the cleavage of pro-apoptotic proteins—CASP3 or PARP (Figure 2C, Supplementary Figure 4B).

### Molecular Modeling Predicts Reduced Binding to the Caseinolytic Mitochondrial Matrix Peptidase Proteolytic Subunit (ClpP) of the Free Base ONC201

Given the predicted pKa values (8.13 and 5.60—by ChemAxon pKa calculator) for the ONC201 basic sites (N1 and N3, respectively), the di-cation is likely to predominate in the upper gut (pH 2), while in blood serum (pH 7.35–7.45), 83% will be represented as the mono-cation, and 17% as the neutral free base (GsONC201). Although the speciation (dihydrochloride salt or free base) should not be influenced by the protonation state of ONC201 when administered orally, we modeled the influence of ONC201 protonation on ClpP and DRD2 binding *in silico*.^17^ Docking analysis indicated that ONC201 speciation influenced binding with ClpP, as the implicitly protonated ONC201 generated higher binding scores (ChemPLP and Goldscore^19^) (Figure 2Di and ii). This suggests that the prevalent mono-protonated species at pH 7.4 has a higher binding affinity for the target than the neutral free base GsONC201. Mono-protonated ONC201 supports several positive interactions, featuring an H-bond between the protonated N1 nitrogen and tyrosine 118, an interaction not seen in either the original co-crystal structure,^10^ or after docking the free base (Figure 2Ci). In contrast, the two different ONC201 species investigated did not show a significant variation in docking scores with DRD2 (Figure 2Dii), despite displaying more diverse binding geometries. Together, this suggests that the DRD2-binding pocket is less specific for ONC201 species.

### PK and PD Analysis Showed Bioavailability and Preclinical Efficacy In Vivo

**Figure 3.**
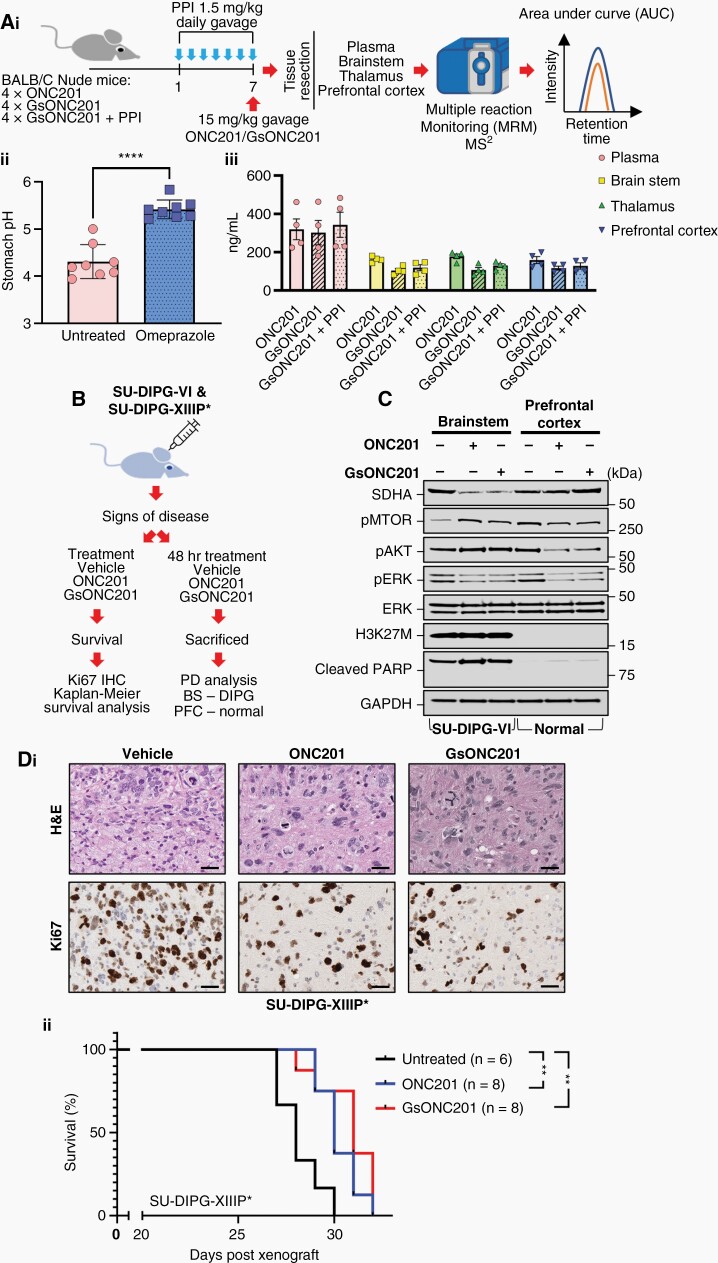
Brain and tumor penetration of ONC201 and German-sourced ONC201 (GsONC201). (Ai) Schematic workflow for pharmacokinetic analysis of ONC201 and GsONC201 ± omeprazole (PPI). (Aii) pH of stomach contents of mice receiving oral 1.5 mg/kg omeprazole daily for 7 days. (Aiii) Multiple reaction monitoring (MRM) analysis of tissue concentrations of Oncoceutics or GsONC201 treated for 1 h prior to sacrifice 15 mg/kg, ± 7 days of prophylaxis administration of omeprazole. Average tissue concentrations; plasma 320.2 ng/mL, brainstem 140.2 ng/mL, thalamus 138 ng/mL, or prefrontal cortex 132 ng/mL. (B) Schematic workflow for pharmacodynamic, histological, and survival analysis of ONC201 and GsONC201. (C) Pharmacodynamic analysis of brainstem H3K27M+ SU-DIPG-VI and control prefrontal cortex tissues following oral treatment with vehicle, ONC201 and GsON201 125 mg/kg and sacrificed 48 h after treatment. (Di) Hematoxylin and eosin staining (H&E) and Ki67 staining of brainstem SU-DIPG-XIIIP* tumor tissue following 2 weeks of treatment with vehicle, ONC201 or GsON201 125 mg/kg. (Dii) Kaplan–Meier survival analysis of SU-DIPG-XIIIP* treated with vehicle, ONC201 or GsON201 125 mg/kg. Median survival of cohorts (days): Vehicle = 28, ONC201 = 31, GsONC201 = 32 (Log-rank [Mantel–Cox] test *p* values vs vehicle *P* = .0065 [95% CI 0.08419–1.2] and *P* = .0029 [95% CI 0.06278–1.042], respectively).

DIPG patients are often prescribed high-dose corticosteroids (eg, dexamethasone) to decrease peritumoral inflammation; however, corticosteroid use is accompanied by adverse effects including gastrointestinal signs and symptoms such as gastritis, dyspepsia, and peptic ulceration, with an increased risk of gastrointestinal hemorrhage.^25^ Addressing these side effects, DIPG patients are commonly prescribed proton pump inhibitors (PPIs), such as omeprazole, to inhibit acid secretion, increasing intragastric pH and thus limiting acid-related adverse effects of corticosteroid therapy.^26^ Indeed, a single oral PPI dose will raise gastric pH in most patients from 2.0 to over 6.0.^27^ Under these elevated gastric pH conditions, the oral absorption of free base GsONC201, which is a weak base, may be decreased due to its insolubility. We therefore assessed the penetration of GsONC201 through the gastric mucosa of mice following the elevation of gastric pH. Mouse brain *in vivo* chemical half-lives are almost identical to human chemical half-lives^28^; therefore, we pretreated mice for 1 week with a clinically relevant dose of omeprazole once per day via gavage, prior to receiving a clinically relevant dose of either ONC201 or GsONC201. One hour following treatment, all mice were sacrificed, and PK analysis was performed using plasma and brain tissues including the brainstem, thalamus, and prefrontal cortex (Figure 3Ai). Treatment with omeprazole for 1 week significantly increased stomach pH levels (Figure 3Aii), however, did not decrease bioavailability of GsONC201 in any of the tissues measured (Figure 3Aiii). These results highlight that even at increased gastric pH levels, GsONC201 showed clinically relevant bioavailability, levels likely to be active in the central nervous system (CNS).

To determine whether GsONC201 has the potential to elicit an anti-DIPG effect in vivo, PD and Kaplan–Meier survival analyses were performed using SU-DIPG-VI-luc and SU-DIPG-XIIIP* orthotopic PDX mouse models treated with either ONC201 or GsONC201 (Figure 3B). PD was determined following 48-h treatment, sacrificed, and brainstem (H3K27M+ tumor) and prefrontal cortex tissues (normal control) resected (Figure 3C). Analogous to* in vitro* results (Figure 2C, Supplementary Figure 4A), both ONC201 and GsON201 decreased the abundance of SDHA, the phosphorylation of ^pT202/Y204^ERK, and ^pS2448^mTOR in both tumor and control tissue compared to vehicle-treated controls (Figure 3C). Contrarywise, increased phosphorylation of ^pS473^AKT was also seen in the tumor tissue, whereas it decreased in the prefrontal cortex, with no evidence of cleaved PARP in either tumor or control tissue (Figure 3C). Histological examination of SU-DIPG-XIIIP* tissue was performed following 2 weeks of treatment with ONC201 or GsONC201. All tissues examined from the brainstem of PDX mice were positive for H3K27M+ staining, whereas the surrounding brainstem tissues were negative (Figure 3Di). Importantly, 2-week treatment of PDX models with either ONC201 or GsONC201 decreased Ki67 staining (Figure 3Dii), however, did not increase the cleavage of PARP and CASP3 (data not shown), analogous to *in vitro* results (Figure 2B–C, Supplementary Figure 4B). Significantly, both ONC201 and GsONC201 extended the survival of PDX models compared to vehicle controls (*P* = .0065 and *P* = .0029, respectively), with no difference between the 2 chemicals (Figure 3Dii).

### Patient Experience Using German-Sourced ONC201

**Figure 4.**
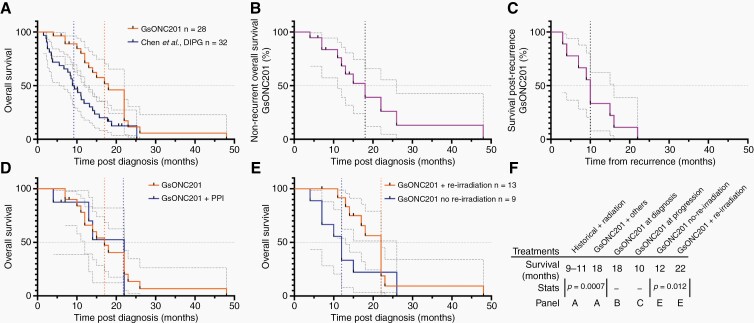
Clinical experience of DIPG patients receiving German-sourced ONC201 (GsONC201). (A) Kaplan–Meier survival analysis reporting median overall survival for all patients receiving GsONC201 compared to brainstem glioma patients reported by Chen et al.^2^ (18 months vs 9.2 months, *P* = .0007 Wilcoxon [Gehan–Breslow] test, 95% CI 1.292–4.153; *P* = .0017 Log-rank [Mantel–Cox] 95% CI 1.447–4.990). (B) Kaplan–Meier survival analysis reporting median overall survival when initiated following initial radiotherapy. (C) Kaplan–Meier survival analysis reporting median recurrent overall survival. (D) Analysis of median overall survival for patients receiving GsONC201 and concomitant proton pump inhibitors (PPI) compared to GsONC201 patients who did not use PPIs (17 vs 22 months, *P* = .7946 Log-rank [Mantel–Cox] test, 95% CI 0.3151–2.507). (E) Kaplan–Meier survival analysis reporting median OS survival for patients who received GsONC201 and underwent reirradiation compared to patients who received GsONC201 and did not have reirradiation (12 vs 22 months, *P* = .0120, Wilcoxon [Gehan–Breslow] test, 95% CI 0.7709–6.143). (F) Summary of survival data.

The median OS of 28 DIPG patients harboring H3K27M mutations who were not enrolled on clinical trials, but receiving GsONC201 between 2017 and 2020, was investigated. Clinical and pharmacological data were assessed, and survival was recorded as of April 20, 2021. Importantly, many other concomitant anticancer and complementary therapies were also used by these patients, therefore survival cannot be attributed to GsONC201 alone (Supplementary Table 1). Median OS for patients who received GsONC201 was 18 months (range 4–48 months) compared to 9–11 months historical records (*P* = .0007, Figure 4A).^2^ For patients who commenced GsONC201 following radiation but prior to recurrence, median OS was also 18 months (n = 19 patients, range 4–48 months) (Figure 4B). An additional 10 months survival was seen for patients initiating GsONC201 at disease progression (n = 6, range 3–16 months) (Figure 4C). The median duration each patient received GsONC201 was 7.5 months (n = 28 patients, range 2–36 months) (Supplementary Table 1). Furthermore, 8 families reported coadministration of PPIs for considerable time periods with GsONC201; however, no statistical difference in survival was seen (PPI 17 months vs no PPI 22 months *P* = .7946) (Figure 4D). An increase in median OS for patients who underwent reirradiation and received GsONC201 was seen compared to patients who received GsONC201 and did not receive reirradiation (22 vs 12 months, *P* = .012) (Figure 4D, Supplementary Figure 1).

## Discussion

At the current time, the outlook for patients diagnosed with DIPG is bleak. The uniformly fatal diagnosis is unacceptable, particularly for parents and their families. Over the course of the last 5 years, several “waves of hope” have been brought forward via social and mainstream media with anecdotal reports of experimental chemicals and treatments, provided outside of the standard of care, providing “benefits”.^29,30^

Numerous preclinical studies have identified potent chemicals that kill DIPG cells in the low nM range^22,31^; however, systemic toxicity, poor bioavailability, and lack of CNS activity have limited their clinical translation as monotherapies with systemic delivery methods. Nevertheless, parents seek access to these chemicals outside of clinical trial protocols. Similarly, a level of uncertainly remains regarding the use of GsONC201. However, differing from other unpublished protocols,^30^ case studies and reports from expanded access programs suggest a therapeutic benefit from Oncoceutics ONC201.^13,32^ PK studies using tissue obtained from patients undergoing re-resection of recurrent glioblastoma showed intratumoral concentrations of ONC201 ~24 h following treatment (625 mg/weekly oral) ranging from 600 nM to 9.3 µM,^32^ highlighting the potential CNS activity and use of the compound for the treatment of DIPG. This has undoubtedly encouraged the synthesis of GsONC201, frequently purchased by parents unable to enroll their children in clinical trials in the United States or Japan or gain expanded access. However, the chemical formulation GsONC201 has hitherto remained unknown.

Herein, analysis of the constituents of GsONC201 capsules confirmed GsONC201 is indeed the active angular isomer (Figure 1, Supplementary Figures 1 and 2A–C) and not the inactive linear isomer that has plagued the use of the chemical.^14,21^ Our NMR analysis identified mannitol as the bulking agent (Figure 1B and C), potentially included as a sweetening agent,^33^ with parents reporting GsONC201 to have a “sweet taste.” Mannitol is also under clinical investigation for the treatment of Parkinson’s disease in a randomized, controlled Phase IIa clinical trial (NCT03823638) and has been used as an intravenous hyperosmolar solution to transiently permeabilize the BBB.^34,35^ It is interesting to postulate that the inclusion of mannitol may be to increase the CNS activity of GsONC201. However, given orally, our PK studies showed no increased CNS penetration in tumor naïve mice when administered via gavage, compared to ONC201 formulated as an acidic salt (Figure 3Aiii).

It is also interesting that GsONC201 is synthesized as the free base rather than the dihydrochloride salt used by Oncoceutics, a feature that limits its water solubility consistent with parents reporting “white precipitants” forming when the contents of GsONC201 capsules are mixed in water. However, given the low pH of the stomach, the protonation state of GsONC201 is inconsequential when administered orally. Indeed, even in situations where the pH of the stomach is elevated (Figure 3Aii), the free base GsONC201 showed similar levels of CNS penetration in mice tissue as that of the ONC201 dihydrochloride salt (Figure 3Aiii). However, if parents were to administer the contents of the GsONC201 capsule in water, it would be almost impossible to deliver the entire contents. Unexpectedly, molecular modeling showed reduced binding of free base GsONC201 to ClpP compared to protonated ONC201. Even though no difference in efficacy was seen *in cellulo*, considerations for the type of formulation may be important if administering ONC201 using local delivery systems such as convection-enhanced delivery.^36^

Previous studies showed that ONC201 has specific anticancer effects through dual inhibition of Akt and ERK, to drive TRAIL expression and potent antitumor effects.^11,14,37^ DIPG cell lines showed varying levels of sensitivity (Figure 2A and B), with highly sensitive cells showing potent Akt and ERK inhibition and cell death (Figure 2C, Supplementary Figure 4A and B). Genome-wide shRNA screens showed neurotransmitter pathways, including the DRD2, are critical to the growth and survival of glioblastoma cell lines (*in vitro* and *in vivo*), highlighting the potential of ONC201/GsONC201, a known DRD2 antagonist.^8,38–40^ The pro-proliferative effects of DRD2 signaling in glioblastoma are mediated, in part, by Ras/ERK signaling, with the use of the antipsychotic haloperidol (FDA approved DRD2 antagonist), like ONC201, used in less sensitive DIPG cells, decreasing ERK activity while having no effect on Akt^38^ (Figure 2C). However, the cellular targets of ONC201 in DIPG remain to be unequivocally determined. Indeed, it is highly possible that in DIPG cells, ONC201 and GsONC201 antagonize DRD2 while simultaneously agonizing ClpP. Independent of the *in vitro* sensitivity of DIPG cell lines, a significant decrease in the phosphorylation of ERK and the abundance of SDHA was seen 72 h after treatment (Figure 2C and Supplementary Figure 4A), with DIPG PDX models also showing a reduced abundance of SDHA and the phosphorylation of ERK highlighting the potential of ONC201 and GsONC201 in preclinical models (Figure 3Ci), and suggestive of ClpP and DRD2 as the dominant cellular targets in DIPG. These complementary effects highlight the therapeutic potential of ONC201* in vivo*.^41^ ONC201 and GsONC201 both modulated mitochondrial function, specifically in DIPG tissues, and potentially decreased global dopamine signaling (Figure 3Ci), to significantly extend the survival of DIPG PDX models, albeit temporarily (Figure 3D). Recent studies show DIPG patients with increased ^18^F-DOPA uptake during MRI, presented a median OS of ≤12 months, and a lower degree of tumor volume reduction following radiotherapy (*P* = .001), independently correlating ^18^F-DOPA uptake with OS.^42^ DOPA is a precursor for dopamine synthesis, potentially implying DIPG can synthesize and secrete dopamine and may play a role in the gliomagenesis of DIPG.^42^ Therefore,* in vivo* DRD2 antagonism both in the tumor and normal tissues (Figure 3Ci) highlights the endogenous and exogenous contributions to the growth and survival of DIPG that we are only beginning to understand.^41^

Importantly, these studies confirm the biological activity of the tested German-sourced GsONC201 with a median OS for patients diagnosed with H3K27M+ DIPG of 18 months (Figure 4A). Patients who received GsONC201 at disease progression survived 10 additional months, which on aggregate exceeds what is reported in recurrent glioblastoma patients who received single-agent weekly oral ONC201 of (6 months), and well exceeds historical recurrent median OS for DIPG patients (1–4 months).^43,44^ Recently, reirradiation for DIPG patients has entered clinical practice, extending median OS advantage by ~3 months.^45^ Comparing patients who underwent reirradiation while receiving GsONC201 showed a significant survival extension compared to those who did not (Figure 4E, Supplementary Table 1).

Although we cautiously make the comparison to historical data,^1,2^ it is very important to note that an array of anticancer and complementary therapeutics were used by patients who received GsONC201, and therefore survival cannot be exclusively attributed to monotherapy—GsONC201. However, it is also important to consider that in historical cases of DIPG, guardians of almost all patients would have also explored alternative, complementary, immunological, or surgical treatments as adjuvants to radiation. The resounding difference reported herein is that patients received GsONC201 for a median duration of 7.5 months (Supplementary Table 1) and survived for 18 months.

We highlight that our report of the structural, biochemical, PK, PD, *in vivo* PDX survival, and patient experience is not presented as a justification for the purchase or the synthesis of GsONC201. Nor do we make the claim that Oncoceutics ONC201 provides a survival benefit and recognize that we must proceed cautiously; until adequate late-stage clinical investigations are completed, it is not possible to confirm the extent of benefit from either Oncoceutics ONC201 or GsONC201 for patients diagnosed with DIPG. However, while ONC201 remains inaccessible to patients outside of the United States and Japan, families will continue to search for experimental options, like GsONC201, sourced internationally with significant associated financial, logistical, and psychosocial costs.

## Supplementary Material

vdab169_suppl_Supplementary_Materials

## Notes

**Authorship statement**: M.D.D., P.A.H., A.M.D., K.E.H., and J.C.M. conceived and designed the study. R.J.D., A.M., T.H., A.S.W., E.R.J., Z.P.G., D.S., I.J.F., N.D.S., P.S.K., and G.N.D. conducted the experiments. M.D.D., J.C.M., A.M.D., T.H., A.S.W., N.D.S., and G.N.D. interpreted the results. P.A.H., K.E.H., J.E.C., B.N., K.I., F.A., J.N., N.A., A.M.L.M., A.B., N.A.V., S.M., and J.C.M. provided discipline-specific knowledge, clinical/patient care, expertise, and patient data. All authors wrote the manuscript. M.D.D., R.J.D., T.H., and G.N.D. designed the figures. All authors discussed and analyzed the results. All authors edited the manuscript and approved the final version.
