# Supplementary material for: Preclinical and clinical evaluation of German-sourced ONC201 for the treatment of H3K27M-mutant diffuse intrinsic pontine glioma
Source: Neurooncol Adv. 2021 Nov 19;3(1):vdab169. doi: 10.1093/noajnl/vdab169 (PMC8709907; doi:10.1093/noajnl/vdab169)

SUPPLEMENTARY INFORMATION

Materials and Methods:

Chemicals and regents

Unless otherwise specified, chemical reagents were obtained from Merck or Thermo Fisher Scientific and were of molecular/mass spectroscopic research grade. All antibodies were purchased from Cell Signaling. HRP-conjugated anti-rabbit IgG was purchased from Sigma.

High-resolution tandem mass spectrometry

High-resolution tandem mass spectrometry was performed on GsONC201 and ONC201 (Oncoceutics) using direct infusion into an accurate mass Thermo Scientific Q Exactive Plus mass spectrometer (Thermo Fisher Scientific). Samples were resuspended in 1 mL of dimethyl sulfoxide (DMSO), diluted 1 in 100 in 50:50 methanol: acetonitrile / 0.1% formic acid and analyzed using a resolution of 140,000 at 200 m/z for both MS1 and MS2. Data analysis was performed using Mass Frontier 7.0 (Thermo Fisher Scientific).

Nuclear magnetic resonance spectroscopy

NMR spectroscopy was performed using either a Bruker Avance 300 (300.13 MHz, ^{1}H; 75.5 MHz, ^{13}C) or an Avance III 400 (400.13 MHz, ^{1}H; 100.6 MHz, ^{13}C) with or without a Prodigy cryoprobe CPPBBO. NMR spectra were processed using TopSpin 3.5 software (Bruker). Chemical shifts are expressed in parts per million (ppm) on the δ scale. Chemical shifts in $CDCl_3$ were referenced relative to $C\mathit{H}Cl_3$ (7.26 ppm) for ^{1}H NMR and $CDCl_3$ (77.16 ppm) for ^{13}C NMR and chemical shifts in $(CH_3)_2SO$ were referenced relative to $(C\mathit{H}_3)_2SO$ (2.50 ppm) for ^{1}H NMR and $(CD_3)_2SO$ (39.52 ppm) for ^{13}C NMR.

Cell culture

The use of DIPG neurosphere cell cultures in this study was approved by the Human Ethics Research Committee, University of Newcastle (H-2018-0241). Patient derived DIPG neurosphere cell lines, SU-DIPG-IV, SU-DIPG-VI, SU-DIPG-XIII, SU-DIPG-XVII, SU-DIPG-XXI, SU-DIPG-XXXVI, were generously donated by Prof. Michelle Monje (Stanford University). DIPG neurosphere cell cultures were maintained in serum-free Tumor Stem Medium (TSM), consisting of 50:50 Neurobasal-A medium and DMEM/F12 with 1% v/v HEPES, 1% v/v Sodium Pyruvate, 1% v/v Non-essential Amino Acids Solution, 1% v/v GlutaMAX-I Supplement and 1% v/v Antibiotic/Antimycotic. At time of use, TSM was supplemented with B27™, Hu-EGF, Hu-FGF, Hu-PDGF-AA, Hu-PDGF-BB, and Heparin and cells passaged using Accutase cell dissociation medium every 1-2 weeks, or when confluent.

Growth and proliferation assays

Cellular growth and proliferation of DIPG cell lines was determined using a resazurin cell proliferation assay.[1] DIPG cells were seeded at 2.5×10^4 cells/well in a 96-well plate and incubated overnight at 37°C. Following neurosphere formation, cells were incubated with a serial dilution of ONC201 (Oncoceutics) or GsONC201, in a total volume of 200 µL. Plates were then incubated for 96 hours. Five hours prior to the endpoint, 20 µL of resazurin was added to each well and incubated for the remaining 5 hours at 37°C, 5% CO_2. Plates were read using a Fluostar system (BMG LABTECH) at 575/585 nm and values graphed as percentage cell proliferation compared to the untreated control.

Apoptosis flow-cytometric assays

To assay apoptosis, DIPG cells were seeded at 2.5×10^4 cells/well in a 96-well plate and incubated overnight at 37°C. Following neurosphere formation, cells were then incubated with 5µM ONC201 (Oncoceutics) or GsONC201, in a total volume of 200 µL for 96h hours. Following treatment, cells were collected, washed three times in cold PBS, and resuspended in 100 µL of Annexin-binding buffer. Cells were stained with 1 µl of Annexin V-FITC antibody and 5 µl of Popidium Iodide (BD

Bioscience) for 15 min in the dark and subsequently were diluted to a total volume of 500uL in Annexin-binding buffer. Fluorescence-activated cell sorting (FACS) analysis was performed on a Facs Canto 2 (BD Bioscience), to a total of 10,000 events for each sample.

Immunoblotting

Western immunoblotting was performed as previously described.[2]

Binding of free base and protonated ONC201 into putative targets

The free base and HCl salt derivative structures of ONC201 were docked into putative targets, CLPP and DRD2 using GOLD Protein Ligand Docking Software (version 2020.2.0).[3,4] The hydrophobic binding pocket for CLPP was defined by the co-crystal structure PDB: 6DL7,[5] whereas the binding pocket for DRD2 was defined by the co-crystal structure of the DRD2 agonist Risperidone PDB: 6CM4.[6] Both ligands were allowed to search their respective conformational spaces during docking simulations. The 10 closest interacting sidechains in each binding pocket were allow to move, to accommodate each docking pose while the remainder of the protein was kept rigid. The top 4 poses for each ligand were recorded and the average fitness scores (ChemPLP, Goldscore) obtained.

Calculation of pKa values

The pKa values of ONC201 (Oncoceutics) or GsONC201 were calculated using the pKa calculator plug-in by ChemAxon as implemented in the MarvinSketch interface, Marvin 17.2.20 (ChemAxon).

Tissue pharmacokinetics and multiple reaction monitoring

The use of animals in this project was approved by the University of Newcastle, Animal Care and Ethics Committee (A-2019-900). ONC201 (Oncoceutics) and GsONC201 were diluted in 1% methylcellulose/0.2% Tween 80. Omeprazole was diluted in phosphate buffer saline (PBS). All drugs and vehicles were administered via gavage to 8-week-old BALB/c Nude mice. Mice were treated

with omeprazole (1.5 mg/kg/daily) for 7 days prior to administration of either ONC201 (Oncoceutics) or GsONC201. After one hour, mice were sacrificed by CO_2 euthanasia. Immediately following euthanasia, blood was extracted via cardiac puncture, and stomachs and brains collected. Blood plasma was separated via standard centrifugation techniques and frozen at -80^oC. Brainstem, thalamus and prefrontal brain regions were dissected prior to snap freezing in liquid nitrogen. Stomach contents were pooled and pH measured using an Orion Star A221 precision pH probe (Thermo Fisher Scientific). Brain tissues were homogenized using Lysing Matrix beads in a FastPrep-24™ 5G system (MP Biomedicals) at the factory recommended settings. ONC201 (Oncoceutics) and GsONC201 were extracted from plasma and homogenized brain tissues using a protein precipitating mixture composed of 90% v/v acetonitrile, 10% v/v ethanol and 0.1% v/v glacial acetic acid. The supernatant was separated and collected following centrifugation. The supernatants were then analyzed using a Nexera X2 UHPLC system (Shimadzu) coupled to a QTRAP 6500 System (SCIEX) via multiple reaction monitoring (MRM) using 268.1 and 164.1 transitions of the 387 m/z precursor mass. Quantitative analysis was conducted using MultiQuant Software (SCIEX) against a calibration curve for ONC201 (Oncoceutics) over the concentration range of 0.061 – 1000.0 pg/µL.

Tissue pharmacodynamics

A single cell suspension of SU-DIPG-VI-Luc neurospheres was injected into the fourth ventricle/pons of BalbC/Nude mice under isoflurane anesthesia. Stereotactic coordinates were 0.8m to the right of midline, 0.5mm posterior to lambda and 5mm deep using 400,000 cells in 2uL. Following signs of successful disease mice were treated with 125mg/kg ONC201 or GsONC201 by gavage in 1% methylcellulose and 0.2% tween 80 for 48-hours before being sacrificed by CO_2 euthanasia. Immediately following euthanasia mice were transcardial perfused with saline, brain removed, brainstem and prefrontal cortex dissected and snap frozen in liquid nitrogen. Tissue was homogenized on ice using a Dounce homogenizer in RIPA protein extraction buffer containing

protease and phosphatase inhibitors. Homogenized samples were sonicated 5x for 20seconds at 4°C, before being clarified through centrifugation at 25,000g for 30minutes at 4°C. Samples were then subjected to standard immunoblotting techniques as previously described.

Orthotopic xenograft mouse models

A single cell suspension of SU-DIPG-XIII-Pons* neurospheres was injected into the fourth ventricle/pons of BalbC/Nude mice under isoflurane anesthesia. Stereotactic coordinates were 0.8m to the right of midline, 0.5mm posterior to lambda and 5mm deep using 400,000 cells in 2uL. SU-DIPG-XIII Pons* xenografted mice were allowed to recover for one week post engraftment before being treated with 125mg/kg ONC201 or GsONC201 by gavage in 1% methylcellulose and 0.2% tween 80, every 5 days for 4 weeks. 24 hours after the last treatment, mice were euthanased via transcardial perfusion with saline, brain removed. Brains were fixed in 10% formalin neutral buffered solution and embedded in paraffin. 5 μm sections were sectioned and mounted on glass slides. Following dehydration, sections were stained with hematoxylin/eosin and Ki67 for histologic examination. Remaining mice were used for survival analysis and analyzed via the Kaplan Meier survival analysis GraphPad Prism Version 9.1.0. was used for in vivo statistical analyses using the Mantel-Cox test. In all cases values of $p<0.05$ were regarded as being statistically significant.

Patient Survival analysis

Kaplan-Meier survival estimates were used for patients receiving GsONC201 (n=28) compared to data published for patients diagnosed with DIPG n=32 (pontine) by Chen *et al.*[7] Log-rank tests were employed to contrast the different subgroups of the variables. Univariate and multivariate Cox proportional hazard regression models were performed to find significant predictors (p values< 0.05) and analyzed using GraphPad Prism Version 9.1.0.

Supplementary Table:

Supplementary Table S1. Clinical information for patients who received German sourced ONC201 (GsONC201) 2017-2021.

Supplementary results captions:

Supplementary Figure S1. ONC201 (Oncoceutics) and German sourced ONC201 (GsONC201) are indistinguishable by high resolution accurate mass, mass spectrometry. Both (**A**) ONC201 (Oncoceutics) and (**B**) GsONC201 exhibited identical precursor ion mass 387.21798^+ m/z. Similarly, fragment ions originating from both chemicals were detected at 105.070^+, 164.082^+, and 268.144^+ m/z matched across three separate samples.

Supplementary Figure S2. Nuclear magnetic resonance (NMR) spectroscopy of authentic sample of ONC201 (Oncoceutics) showed that it is formulated as the dihydrochloride salt and converting the purified active component of German sourced ONC201 (GsONC201) to the dihydrochloride salt gives a matching spectrum. (A) ^{1}H NMR spectrum of ONC201 (Oncoceutics) in DMSO-d6 as the dihydrochloride salt. **(B)** ^{1}H NMR spectrum of purified GsONC201 in DMSO-d6 after being converted to the dihydrochloride salt by stirring with an excess of a methanolic HCl solution.

Supplementary Figure S3. Nuclear magnetic resonance (NMR) spectroscopy of German sourced ONC201 (GsONC201) shows that it is formulated as the free base and that converting ONC201 (Oncoceutics) to the free base gives a matching spectrum, with both spectra matching the literature values reported for this compound.[8,9] (**A**) ^{1}H NMR spectrum of soluble component of GsONC201 capsule in $CDCl_3$ formulated as the free base. (**B**) ^{1}H NMR spectrum of ONC201 (Oncoceutics) in $CDCl_3$ after being converted to the free base by washing the sample with an aqueous solution of $NaHCO_3$.

Supplementary Figure S4. Biochemical analysis of cellular effects of ONC201 (Oncoceutics) and German sourced ONC201 (GsONC201). (**A**) Immunoblots of signaling pathway phosphorylation and protein expression using ONC201 -sensitive (SU-DIPG-XXI) and -resistant (SU-DIPG-VI and SU-DIPG-XIII) treated with 5 μM ONC201 or GsONC201 following 72-hours treatment (representative immunoblots presented, n = minimum of 3). (**B**) Both ONC201 (Oncoceutics) and GsONC201 drive programmed cell death pathways in sensitive DIPG cell lines.

Supplementary References:

1. Murray HC, Enjeti AK, Kahl RGS, et al: Quantitative phosphoproteomics uncovers synergy between DNA-PK and FLT3 inhibitors in acute myeloid leukaemia. Leukemia, 2020
2. Dun MD, Anderson AL, Bromfield EG, et al: Investigation of the expression and functional significance of the novel mouse sperm protein, a disintegrin and metalloprotease with thrombospondin type 1 motifs number 10 (ADAMTS10). Int J Androl 35:572-89, 2012
3. Jones G, Willett P, Glen RC, et al: Development and validation of a genetic algorithm for flexible docking. J Mol Biol 267:727-48, 1997
4. Verdonk ML, Cole JC, Hartshorn MJ, et al: Improved protein-ligand docking using GOLD. Proteins 52:609-23, 2003
5. Ishizawa J, Zarabi SF, Davis RE, et al: Mitochondrial ClpP-Mediated Proteolysis Induces Selective Cancer Cell Lethality. Cancer Cell 35:721-737 e9, 2019
6. Wang S, Che T, Levit A, et al: Structure of the D2 dopamine receptor bound to the atypical antipsychotic drug risperidone. Nature 555:269-273, 2018
7. Chen LH, Pan C, Diplas BH, et al: The integrated genomic and epigenomic landscape of brainstem glioma. Nat Commun 11:3077, 2020
8. Jacob NT, Lockner JW, Kravchenko VV, et al: Pharmacophore reassignment for induction of the immunosurveillance cytokine TRAIL. Angew Chem Int Ed Engl 53:6628-31, 2014

9. Wagner J, Kline CL, Pottorf RS, et al: The angular structure of ONC201, a TRAIL pathway-inducing compound, determines its potent anti-cancer activity. Oncotarget 5:12728-37, 2014

Patient ID	Diagnosis Date	Age at Diagnosis (years months)	Sex	Mutation status (if known)	Time from diagnosis to deONC201 (months)	Started deONC201 @ recurrent disease (Y/N)	Dose* (mg/kg weekly or total weekly dose)	Duration of therapy** (months)	Concurrent anti-cancer therapies***	Concurrent non anti-cancer therapies***	Patient Succumbed (Y/N)	Event Free Survival (months)	Recurrent Survival (months)	Re-irradiation? (Y/N) or NA	Current Survival** (months)	Overall Survival (months)
CSRG1	17/02/2018	2y 9m	F	H3.1K27M PIK3CA, BCOR, ACVR1, PTEN-loss, VEGF OE	13	Y	15	9	bevacizumab (14 months), sirolimus (3 months), paxalisib (12 months), vandatenib (3 months), panobinostat (6 doses)	CBD	Y	NA	9	Y	NA	22
CSRG2	26/09/2018	3y 2m	F	H3.3K27M	8	N	15	18	paxalisib (1 month)	CBD	Y	24	NA	N	NA	26
CSRG3	8/05/2019	12y 0m	F	H3.3K27M PI3KR1, ATRX loss, PPM1D mutation, low BRCA1 expression	3	N	624mg total	19	everolimus	pantoprazole[#]	Y	14	8	Y	NA	22
CSRG4	unreported	6y 5m	M	H3.3K27M	4	N	20	8	ribociclib, everolimus	pantoprazole[#], loratadine, metformin, melatonin, valproate	Y	9	4	Y	NA	13
CSRG5	28/05/2018	13y	M	H3.1K27M	3	N	635mg total	15	immunotherapy, sirolimus	unreported	Y	11	7	Y	NA	18
CSRG6	Nov-19	6y 1m	F	H3.1K27M	4	Y	312mg total	10	paxalisib, MTX110 via CED, sirolimus	lansoprazole[#], TBL12	Y	NA	3	Y	NA	14
CSRG7	15/06/2018	14y 3m	M	H3.3K27M PTEN-loss	6	Y	11	16	paxalisib	omeprazole[#], CBD	Y	6	16	Y	NA	22
CSRG8	approx 07/09/2020	7y	M	H3.3K27M	4	N	468mg total	2**	bevacizumab	dexmethasone, omperazole[#]	N	NR	NR	NA	6	NR
CSRG9	unreported	3y	F	H3.3K27M	15	Y	312mg total	7	bevacizumab	CBD	Y	NA	7	unreported	NA	22
CSRG10	approx 11/08/2020	8y	M	H3.1K27M	3	N	312mg total	4**	bevacizumab	CBD, levetiracetam, dexamethasone, famotidine[#]	N	NR	NR	NA	7	NR
CSRG11	approx 30/01/2020	5y	F	H3.1K27M	12	N	312mg total	2**	unreported	unreported	N	NR	NR	NA	14	NR
CSRG12	5/05/2020	6y 4m	F	H3.3K27M	2	N	13	3	NIL	CBD, lansoprazole[#]	Y	NA	NA	N	NA	4
CSRG13	approx 01/07/2020	7y 2m	F	H3.1K27M, ACVR1, PIK3CA, FGFR3, MSH6	2	N	312mg total	6**	unreported	CBD	N	NA	NR	NA	8	NR
CSRG14	19/03/2017	5y7m	F	H3.3K27M , ATM, NOTCH2, loss of 16q	12	N	30	36**	immunotherapy, sirolimus	THC+CBD, TBL12, propranolol, fenofibrate,	Y	34	14	Y	NA	48
CSRG15	16/10/2019	7y 4m	M	H3.3K27M,TP53	0.5	N	22	6	bevacizamab	10THC:10 CBD, boswellia serrata extract with 20% AKBA, curcumin, TBL-12, melatonin, liposomal vitamin C, vitamin D3, vitamin K2, vitamin A, selen 12, omega 3, co-Q10, probiotic, magnesium	Y	5	unreported	N	NA	7
CSRG16	8/08/2020	15y 1m	M	H3.3K27M, TP53, PIK3R1, ATRX	<1	N	510mg total	6**	bevacizumab	steroids, levetiracetam	N	NR	NR	NA	7	NR
CSRG17	9/04/2018	3y 1m	M	H3.1K27M	13	Y	19.5	4	nivolumab, dendritic cell vaccine	dronabinol (THC), CBD, valproate, metformin, dexamethasone, Optune therapy	Y	11	4	Y	NA	17
CSRG18	17/10/2018	4y 1m	F	H3K27M, H3C2, PIK3CA, ACVR1	14	Y	24.7	7	temozolomide, everolimus (only 5 days)	frankincense, dexamethasone, trimethoprim / sulfamethoxazole, amphoteracin B, ondansetron	Y	11	8.5	Y	NA	22
CSRG19	11/09/2019	6y 11m	F	H3K27M	2	N	312mg total	6	regorafenib	unreported	Y	7	4	Y	NA	11
CSRG20	9/09/2016	3 y 11m	M	H3.3K27M	1	N	468mg total	4	temozolomide	dexamethasone, valproate	N	4	NA	Y	7	NR
CSRG21	14/04/2020	12y 3m	M	H3K27M	11 weeks	N	340mg total	9	temozolomide	Sativex (nabiximols)	Y	10	2	N	NA	12
CSRG22	4/12/2017	7y 5m	F	H3K27M	16	Y	156mg total	3	ponatinib, temozolomide	CBD, THC, francincence, vitamin D, Q10, selenium	Y	6	13	Y	NA	23
CSRG23	7/05/2018	8y 10m	M	H3.3K27M, LMNB1, TP53, ZMIZ,	7	Y	90	3	ipilimumab, nivolumab	CBD, THC, francincence,	Y	0	10	N	NA	10
CSRG24	23/01/2020	5y 6m	F	H3K27M	3	N	312mg total	10	bevacizumab	CBD, THC, vitamin D, vitamin K, vitamin C, omega 3 oil, LaVita Supplement	Y	7	5	N	NA	15
CSRG25	21/03/2019	8y 4m	F	H3K27M	8	Y	13	3		CBD, THC	Y	6.5	3	N	NA	12
CSRG26	11/02/2020	4y 9m	F	H3K27M	4	N	156mg total	10	temozolomide	Sativex (nabixamols), vitamin D, phosphorus, valproate, trimethoprim/sulfamethoxazole	N	14	NA	N	15	NR
CSRG27	30/01/2020	6y 6m	F	H3.3K27M	3	N	15	12	temozolomide	trimethoprim/sulfamethoxazole, vitamin D3, valproate, CBD, nordic oil, boswellia serrata, pregabalin, omeprazole[#], dexamethasone, Laxbene Junior laxative, ondansetron, bisacodyl, simethicone/dimethicone, fentanyl	N	5	NA	Y	15	NR
CSRG28	29/01/2020	4y 7m	M	H3K27M	3	N	15	3	temozolomide	boswellia, ibuprofen	Y	4	1	N	NA	7

* regimen for some patients included twice weekly dosing - reported values are averaged to weekly mg/kg amounts where patient bodyweight reported
** for living patient at March 2021
*** concurrent at any point throughout deONC201 therapy
"NA" Not Applicable
"NR" Not Reached
"OE" Over Expressed
[#] proton pump inhibitor (PPI)

Supplementary Figure S1.

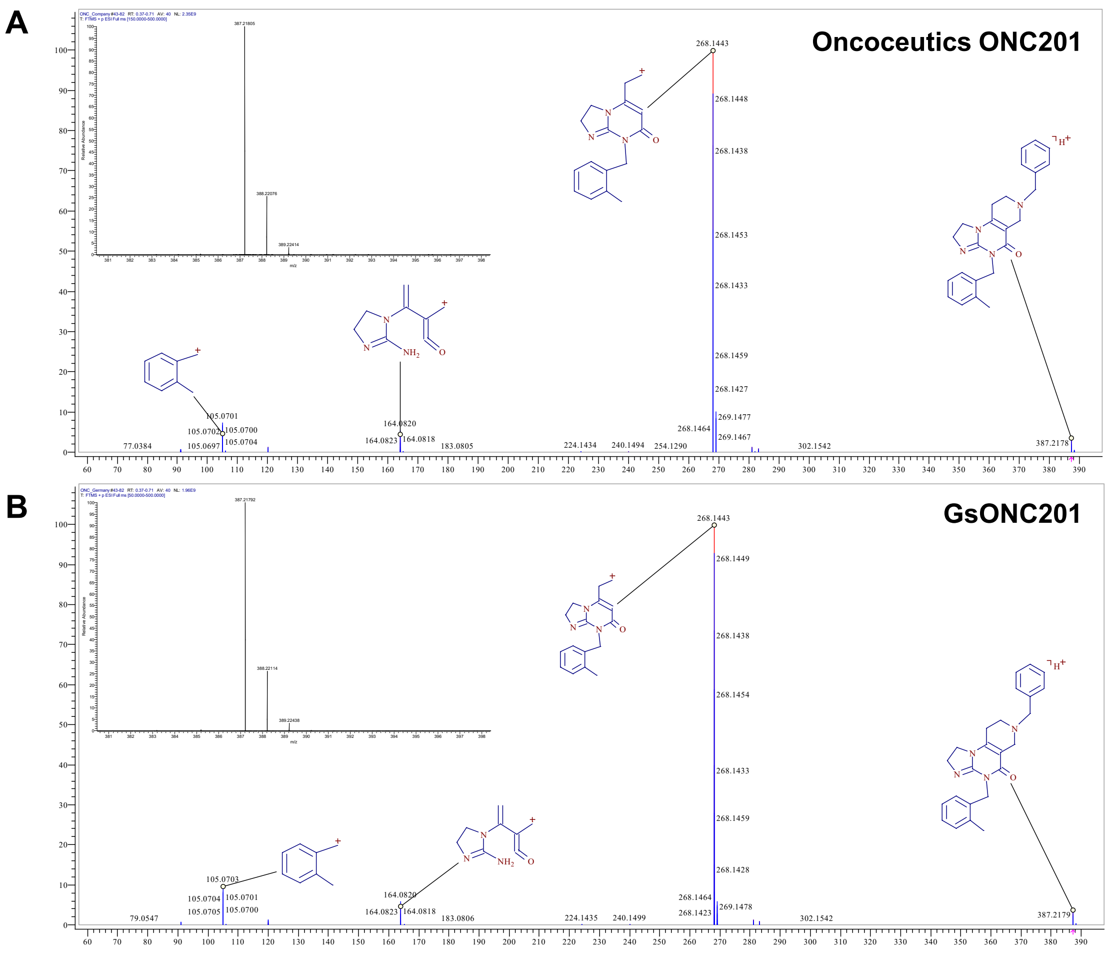

Supplementary Figure S2.

A ^1H NMR spectrum of authentic ONC201 in DMSO-d6

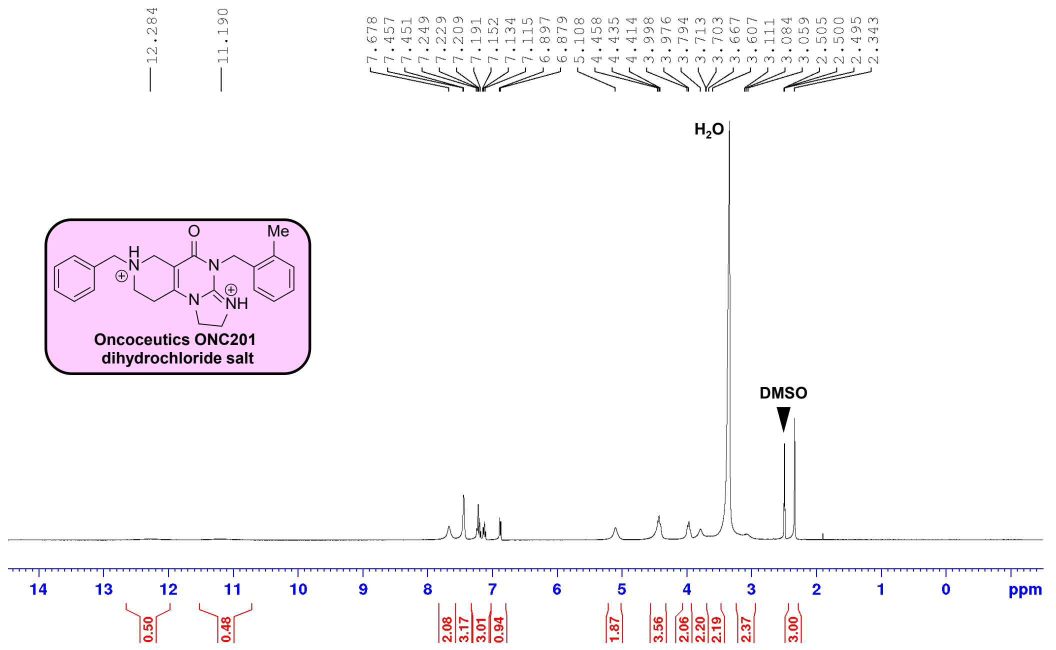

B ^1H NMR spectrum of GsONC201 in DMSO-d6 after being converted to the dihydrochloride salt

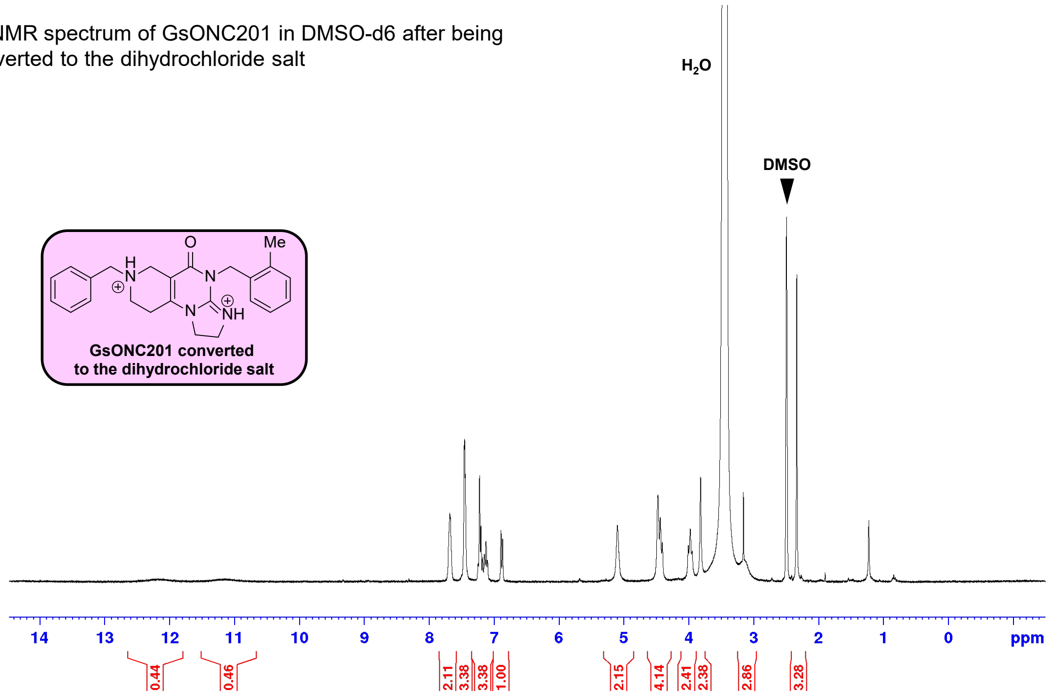

Supplementary Figure S3.

A ^1H NMR spectrum of GsONC201 in $CDCl_3$

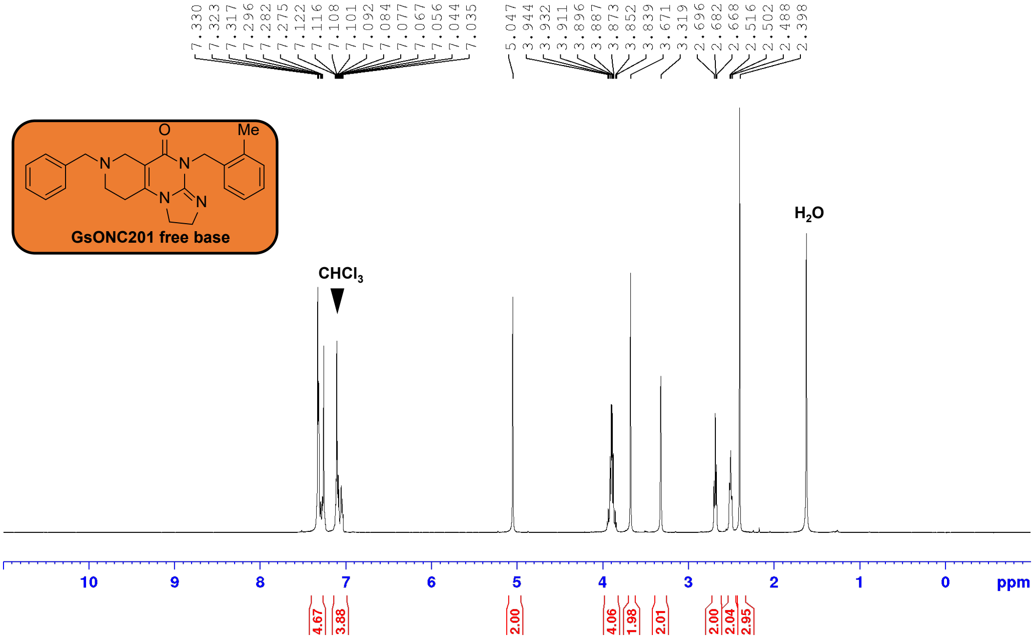

B ^1H NMR spectrum of Oncoceutics ONC201 in $CDCl_3$ after being converted to the free base

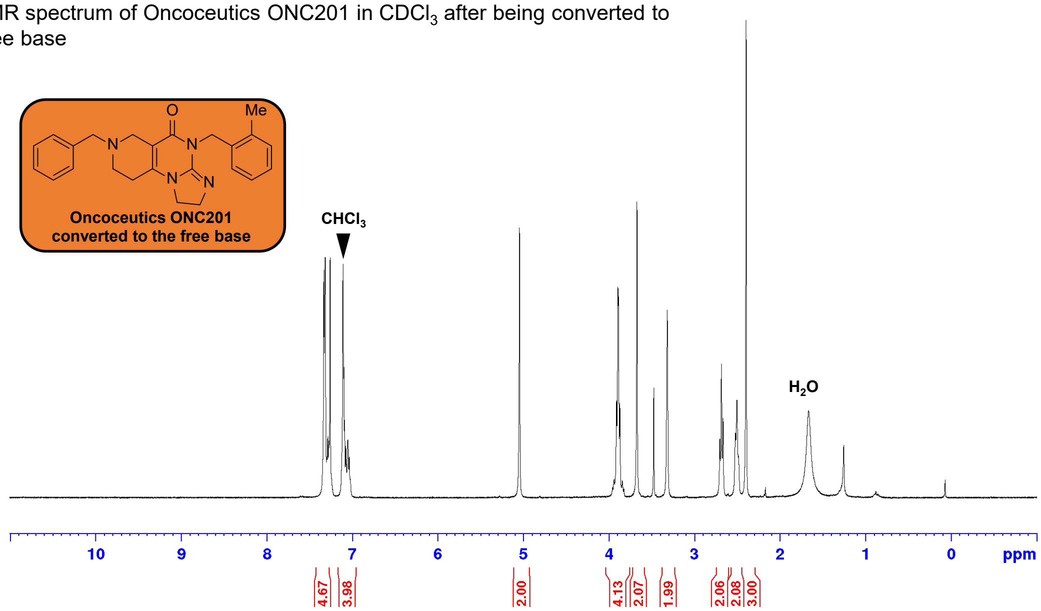

Supplementary Figure S4.

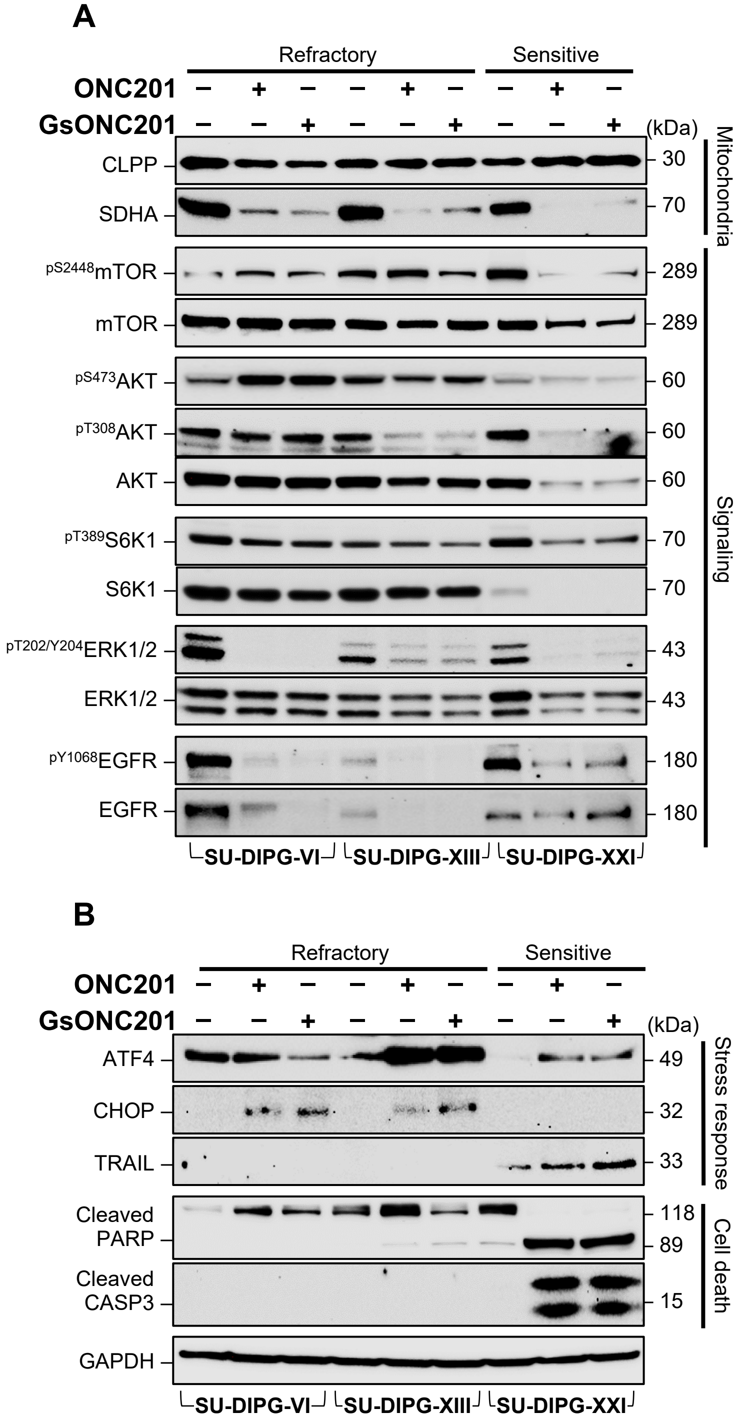

Supplement: vdab169_suppl_Supplementary_Materials [file vdab169_suppl_supplementary_materials.pdf]
